# Association of Scrub Typhus in Children with Acute Encephalitis Syndrome and Meningoencephalitis, Southern India

**DOI:** 10.3201/eid2904.221157

**Published:** 2023-04

**Authors:** Tina Damodar, Bhagteshwar Singh, Namratha Prabhu, Srilatha Marate, Vykuntaraju K. Gowda, A.V. Lalitha, Fulton Sebastian Dsouza, Sushma Veeranna Sajjan, Mallesh Kariyappa, Uddhava V. Kinhal, P.V. Prathyusha, Anita Desai, Kandavel Thennarasu, Tom Solomon, Vasanthapuram Ravi, Ravi Yadav

**Affiliations:** National Institute of Mental Health and Neurosciences, Bangalore, India (T. Damodar, N. Prabhu, S. Marate, P.V. Prathyusha, A. Desai, K. Thennarasu, V. Ravi, R. Yadav);; Christian Medical College, Vellore, India (B. Singh);; University of Liverpool, Liverpool, UK (B. Singh, T. Solomon);; Liverpool University Hospitals NHS Foundation Trust, Liverpool (B. Singh, T. Solomon);; Indira Gandhi Institute of Child Health, Bangalore (V.K. Gowda, U.V. Kinhal);; St. John’s Medical College and Hospital, Bangalore (A.V. Lalitha, F.S. Dsouza);; Vani Vilas Women and Children’s Hospital, Bangalore Medical College and Research Institute, Bangalore (S.V. Sajjan, M. Kariyappa);; The Walton Centre NHS Foundation Trust, Liverpool (T. Solomon);; The Pandemic Institute, Liverpool (T. Solomon)

**Keywords:** scrub typhus, acute febrile encephalopathy, central nervous system infections, encephalitis, *Orientia tsutsugamushi*, vector-borne infections, meningitis/encephalitis, India, bacteria

## Abstract

Scrub typhus is an established cause of acute encephalitis syndrome (AES) in northern states of India. We systematically investigated 376 children with AES in southern India, using a stepwise diagnostic strategy for the causative agent of scrub typhus, *Orientia tsutsugamushi*, including IgM and PCR testing of blood and cerebrospinal fluid (CSF) to grade its association with AES. We diagnosed scrub typhus in 87 (23%) children; of those, association with AES was confirmed in 16 (18%) cases, probable in 55 (63%), and possible in 16 (18%). IgM detection in CSF had a sensitivity of 93% and specificity of 82% compared with PCR. Our findings suggest scrub typhus as an emerging common treatable cause of AES in children in southern India and highlight the importance of routine testing for scrub typhus in diagnostic algorithms. Our results also suggest the potential promise of IgM screening of CSF for diagnosis of AES resulting from scrub typhus.

Scrub typhus is an acute febrile illness caused by an obligate intracellular gram-negative bacterium, *Orientia tsutsugamushi*. It is transmitted through chigger mites and is considered endemic to the tsutsugamushi triangle (covering Asia, northern Australia, and islands in the Indian and Pacific Oceans), although scrub typhus caused by other *Orientia* species has also been reported in Africa, France, the Middle East, and South America (*1*). A recent systematic review from hospital-based studies in India reported 25% of acute undifferentiated febrile illness was caused by scrub typhus. Most studies included were from southern India, but only 20% of included patients were <15 years of age (*2*). Although scrub typhus illness is typically self-limiting, neurologic complications are seen in 20%–25% of patients admitted to the hospital and are associated with high mortality rates (*3*,*4*). Scrub typhus can result in myriad neurologic manifestations, including meningitis, meningoencephalitis, encephalopathy, seizures, stroke, neuropathy, optic neuritis, myositis, myelitis, involuntary movements, and Guillain-Barré syndrome, all of which are well recognized in adults (*3*,*4*).

Recent studies in India have identified *O*. *tsutsugamushi* as a major cause of acute encephalitis syndrome (AES) outbreaks, especially in northern states of the country, such as Uttar Pradesh, Bihar, West Bengal, and Assam (*5*–*7*). Outbreaks of AES pose a major public health problem in India, predominantly affecting children (*8*). The definition of AES used for syndromic surveillance is broad and includes all patients experiencing acute onset of fever and altered mental state (*9*,*10*). The clinical manifestation might be caused by encephalitis or meningitis (direct invasion of the central nervous system [CNS] by the pathogen) or encephalopathy without CNS invasion, such as in the case of severe systemic infection, metabolic derangement, or other neurologic complications after the infection (*10*,*11*). Identifying the pathogenesis could inform management and prognosis (*10*,*12*).

Early diagnosis is key to initiating prompt specific treatment, which can reduce complications and fatality rates of scrub typhus (*2*,*13*). Clinical diagnosis can be challenging because of the overlap of symptoms with other tropical infections endemic to the area that can also cause AES (*5*), such as dengue, chikungunya, malaria, and leptospirosis (*14*). Current microbiological diagnostics for scrub typhus, which are usually based on detecting IgM in serum samples or nucleic acid by PCR, have limitations. IgM appears in serum 5–6 days after onset of illness, can persist long after acute illness, and might cross-react with IgM of other cocirculating pathogens (*14*,*15*). Therefore, in AES patients with simultaneous microbiological evidence for another potential pathogen and *O*. *tsutsugamushi*, confirming *O*. *tsutsugamushi* as the cause is difficult. Detection of IgM in cerebrospinal fluid (CSF) is yet to be used widely in patients with suspected neurologic scrub typhus. Immunofluorescence assay has long been considered the reference standard serologic test, but its use is limited by expense and challenges in interpretation. PCR might help overcome shortcomings of serologic tests with respect to cross-reacting and persisting antibodies, but a positive result is only likely during the bacteremia phase of infection (*16*). Moreover, the recommended samples for *O*. *tsutsugamushi* PCR are blood or eschar material, whereas the sensitivity of PCR on CSF remains unclear (*7*,*16*,*17*). Therefore, a diagnostic approach using accessible tests to determine the association of scrub typhus with AES is urgently needed.

We present preliminary findings of an ongoing multicenter prospective cohort study suggesting scrub typhus as a cause of AES in children in southern India. We used a diagnostic strategy to investigate the association of scrub typhus with AES. We describe the clinical spectrum, epidemiology, and laboratory findings of children with scrub typhus manifesting as AES. We then identify patients demonstrating evidence of meningoencephalitis or encephalitis and explore the value of performing IgM ELISA on CSF samples.

## Methods

### Patients and Study Sites

We prospectively enrolled pediatric patients from 1 month to 18 years of age who fulfilled the Indian National Vector Borne Disease Control Programme (NVBDCP) and World Health Organization case definition of AES (*8*) (Appendix Table 1) and with illness duration of <30 days at the time of hospital admission. Patients were those treated at 3 tertiary-care hospitals in Bangalore, Karnataka state, India (Indira Gandhi Institute of Child Health, St. John’s Medical College and Hospital, and Vani Vilas Hospital), during March 2019–March 2022.

### Ethics Statement

The study was approved by the institutional ethics and review boards of the hospitals and the coordinating center, National Institute of Mental Health and Neurosciences. Full informed consent was taken by the study team, who were trained specifically in taking consent from caregivers, and assent from older children, using procedures and forms approved by the institutional ethics committees.

### Clinical Assessment and Data Collection

Clinical coinvestigators (V.K.G., L.A.V., F.S.D., S.S., M.K.) from the 3 centers performed clinical and neurologic examination of patients. After obtaining consent, we entered detailed clinical history and examination findings on an electronic clinical proforma. Results of routine laboratory tests and patient demographics were collected and entered online by N.P., S.M., or T.D. We determined the normal range of routine laboratory tests according to the age of the patient (*18*) and defined single-organ dysfunction and multiorgan dysfunction syndrome according to established criteria (*19*).

### Microbiological Testing

**Figure 1:**
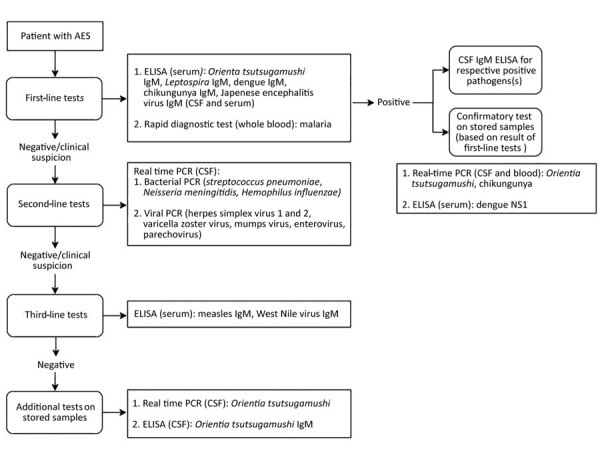
Laboratory algorithm used for serologic and molecular testing of samples from children with acute encephalitis syndrome, southern India. In case of clinical suspicion of a second-line pathogen, irrespective of the first line test results, second-line tests were performed. Similarly, in the case of clinical suspicion of a third-line pathogen, third-line tests were performed. AES, acute encephalitis syndrome; CSF cerebrospinal fluid.

Blood and CSF specimens of enrolled patients were tested at the Department of Neurovirology, National Institute of Mental Health and Neurosciences, by using a laboratory algorithm designed by Ravi et al. (*5*) with some modifications (Figure 1). First-line tests included serum IgM ELISA for various pathogens. CSF samples of patients with IgM-positive ELISA serum samples were diluted in 1:10 proportion for detection of IgM. We performed confirmatory tests on IgM-positive patients, including real-time PCR for *O*. *tsutsugamushi* on CSF and blood samples. For PCR, we extracted DNA from samples by using the QIAamp DNA mini kit (QIAGEN, https://www.qiagen.com) and performed real-time PCR targeting the 47kDa protein gene using the protocol described by Jiang et al. (*20*). In addition, we also performed real-time PCR and IgM ELISA for *O*. *tsutsugamushi* on stored CSF samples of patients with a negative result after third-line tests. We used the Scrub Typhus Detect IgM ELISA kit (InBios International, http://inbios.com) and considered an optical density (OD) cutoff of 0.8 in serum (*15*) and 0.5 in CSF (*21*) samples to be positive. Scrub typhus was diagnosed in patients with IgM-positive real-time PCR or ELISA.

The level of certainty of association of scrub typhus with AES in cases positive for >1 microbiological test(s) for *O*. *tsutsugamushi* was determined by using criteria determined by Granerod et al. (*11*) with modifications (Tables 1, 2). We identified patients with meningoencephalitis/encephalitis (ME) and scrub typhus ME as those demonstrating clinical signs of either encephalitis or meningoencephalitis (Table 2).

**Table 1:** Diagnostic criteria for certainty in the association of AES with scrub typhus in children, southern India*

Association of AES with scrub typhus	Real-time PCR	Serum IgM ELISA	CSF IgM ELISA	Simultaneous evidence of another pathogen(s)
Confirmed	+	+/−	+/−	+/−
Probable, single†	–	+	+/−	–
Probable, co-positive‡	–	+	+	+
Possible	–	+	–	+

*AES, acute encephalitis syndrome; CSF, cerebrospinal fluid. †Without evidence of another potentially causative pathogen. ‡With evidence of another potentially causative pathogen.

**Table 2:** Definitions used for diagnostic association of AES with scrub typhus, ME, and scrub typhus ME in study of association of AES with scrub typhus in children, southern India*

Condition	Definition
Diagnostic association of AES with scrub typhus	Confirmed: Detection of *Orientia tsutsugamushi* DNA by PCR in CSF or blood
	Probable (single): Positive serum IgM ELISA with or without positive CSF IgM ELISA for *Orientia tsutsugamushi* and no other explanatory pathogen or cause
	Probable (co-positive): Positive serum IgM ELISA and positive CSF IgM ELISA for *Orientia tsutsugamushi* with evidence of another pathogen(s)
	Possible: Positive serum IgM ELISA and negative CSF IgM ELISA for *Orientia tsutsugamushi* with evidence of another pathogen(s)
Meningoencephalitis (*22*)	Presence of >1 of the following findings: CSF pleocytosis, meningeal enhancement or parenchymal inflammation on contrast enhanced CT or MRI of brain, positive real time PCR in CSF
Scrub typhus ME	Patients with meningoencephalitis AND positive real-time PCR or serum IgM ELISA for *Orientia tsutsugamushi* (and no other explanatory pathogen or cause) (i.e, patients with confirmed or probable [single] diagnostic association of AES with scrub typhus)

*AES, acute encephalitis syndrome; CSF, cerebrospinal fluid; CT, computed tomography; ME, meningoencephalitis; MRI, magnetic resonance imaging.

### Statistical Analysis

We performed statistical analysis by using R version 3.6.3 (The R Project for Statistical Computing, https://www.r-project.org). We presented descriptive data for categorical variables as frequencies, percentages, or both and described continuous variables using mean +SD or median and interquartile range (IQR). To describe the diagnostic accuracy of CSF IgM, we compared results against CSF PCR to calculate the sensitivity, specificity, positive predictive value (PPV), and negative predictive value (NPV) of CSF IgM with 95% CI. We also calculated those values for patients with scrub typhus ME.

## Results

We included a total of 376 children with AES in the study (Appendix Figure 1). Of those, scrub typhus was diagnosed in 87 patients by using the laboratory algorithm described.

### Microbiological Testing

We collected samples for microbiological testing a median of 11 (IQR 8–14) days from onset of symptoms and median of 4 (IQR 2–6) days after hospitalization. Serum samples were positive for *O. tsutsugamushi* IgM in 86/376 (22.8%) patients. Of those 86 patients, 39 (45.4%) had a positive microbiological test result for another pathogen (referred to as copositive) (Appendix Table 2); 47 (54.6%) were positive for *O. tsutsugamushi* IgM alone (referred to as single-positive). 

CSF samples were available for 82/86 patients with *O. tsutsugamushi* IgM–positive serum samples and all 184 patients who had no etiologic diagnosis after use of the laboratory algorithm. CSF samples were IgM-positive in 58/82 (71%) patients (23/36 of copositive patients and 35/45 of single-positive patients). All 184 serum IgM-seronegative patients were negative for CSF IgM by ELISA.

**Figure 2:**
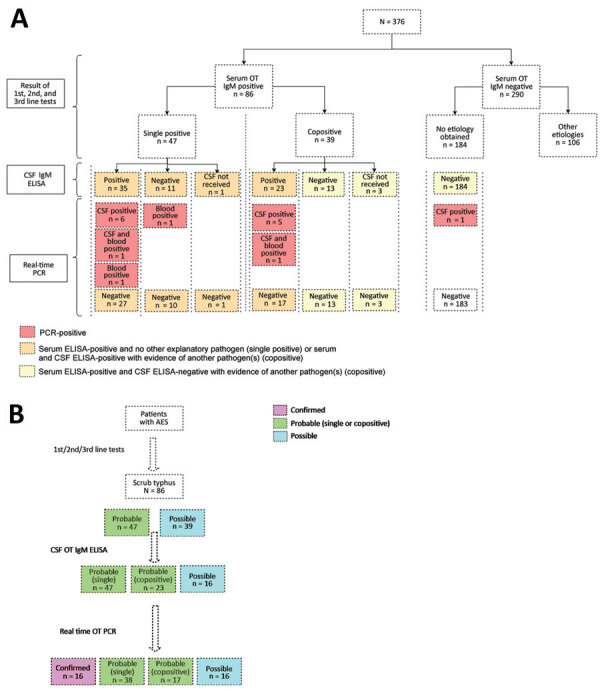
Results of microbiological tests for scrub typhus (A) and diagnostic association of scrub typhus with acute encephalitis syndrome (B) in children, southern India. AES, acute encephalitis syndrome; CSF, cerebrospinal fluid; OT, *Orientia tsutsugamushi*.

Real-time PCR results were positive in 15/86 (17%) patients with IgM-positive serum (real-time PCR of both CSF and blood was positive in 2 patients; 11 were positive by CSF PCR only and 2 by blood PCR only). Of the 184 CSF samples of patients with no etiologic diagnosis after first-line and second-line tests, 1 was positive by real-time PCR for *O. tsutsugamushi*. In total, 16 patients were positive for *O. tsutsugamushi* by PCR. Therefore, of 376 patients with AES, 87 (23%) had a positive microbiological test for scrub typhus (AES–scrub typhus) (Figure 2). 

### Diagnostic Association of Scrub Typhus with AES 

On the basis of serum IgM results, the association of scrub typhus with AES was probable in 47/87 (54%) patients and possible in 39/87 (45%) patients. Further, on performing IgM ELISA on CSF samples, the association was probable (single-positive) in 47 (58.8%) persons, probable (copositive) in 23 (26.4%) persons, and possible in 16 (18%) persons. Finally, on the basis of real-time PCR results, the association was confirmed in 16 (18%) patients, probable (single-positive) in 38 (43.7%) patients, probable (copositive) in 17 (19.5%) patients, and possible in 16 (18.4%) patients (Figure 2).

### ME and Scrub Typhus ME

**Figure 3:**
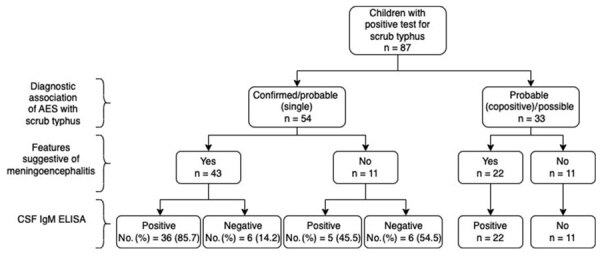
CSF IgM ELISA results of children with scrub typhus ME, southern India. CSF samples were available in 42/43 children with ME of which CSF IgM was positive in 85.7% children. AES, acute encephalitis syndrome; CSF, cerebrospinal fluid; ME, meningoencephalitis.

Of the 87 patients, 65 (74.7%) had findings suggestive of ME (Appendix Tables 3, 4). The diagnostic association of ME with scrub typhus was confirmed or probable (single-positive) in 54 (62%) patients (Figure 2), and of those patients, 43 had ME. Therefore, among all 87 patients, 49.4% had scrub typhus ME (Figure 3).

### Diagnostic Accuracy of CSF IgM Testing 

We performed IgM ELISA and real-time PCR on CSF samples of 266 patients (i.e., 82/86 patients with IgM-positive serum samples and 184/184 patients with no etiology after tests were performed per the laboratory algorithm). We created a 2×2 table to compare the performance of CSF IgM with CSF PCR. The sensitivity of CSF IgM ELISA was 92.9% (95% CI 66.1%–99.8%), specificity 82.1% (95% CI 76.8%–86.6%), PPV 22.4% (12.5%–35.2%), and NPV 99.5% (97.3%–100%) (Table 3).

**Table 3:** Performance of cerebrospinal fluid IgM ELISA compared with cerebrospinal fluid real-time PCR for scrub typhus in children with acute encephalitis syndrome and meningoencephalitis, southern India

Cerebrospinal fluid IgM	Cerebrospinal fluid PCR		
	No. (%) positive	No. (%) negative	Total
Positive	13 (92.8)	45 (17.8)	58
Negative	1 (7.1)	207 (82.1)	208
Total	14	252	266

CSF samples were available for 53/54 patients with confirmed or probable (single-positive) scrub typhus. CSF IgM was positive in 36/42 (85.7%) patients with ME and 5/11 (45.5%) patients without ME. Sensitivity of CSF IgM in patients with ME was 85.7% (95% CI 71.4–94.5%) and specificity was 54.5% (95% CI 23.3%–83.2%); the corresponding PPV was 87.8% (78.8%–93.3%) and NPV was 50.0% (28.6%–71.4%) (Figure 3).

### Demographic and Clinical Profile

**Figure 4:**
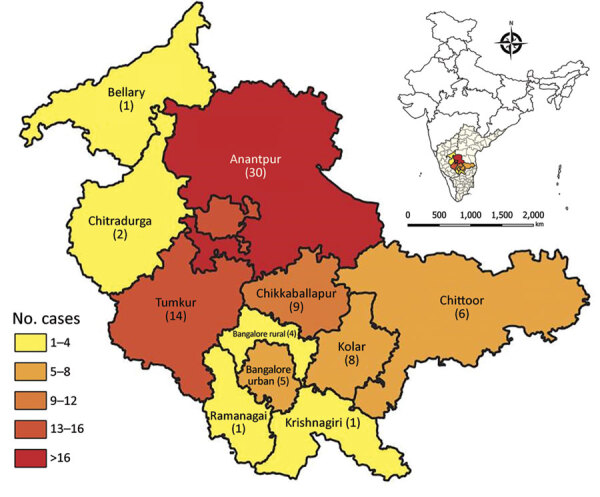
District-wise distribution of children with scrub typhus manifesting as acute encephalitis syndrome, southern India. Number of children from each district with scrub typhus manifesting as acute encephalitis syndrome is indicated. The 3 recruiting hospitals and the coordinating center are located in Bangalore urban. Inset shows location of study area in India.

The male:female ratio of children with scrub typhus was 1.5:1. Ages ranged from 2 months to 17 years; the mean age was 8.5 (SD +4) years (Table 4). Proportions of AES-scrub typhus cases were highest in the months of August and September. In addition, the number of AES-scrub typhus patients and their proportion of total AES patients followed the same pattern as the total number of AES cases (Appendix Figures 2, 3). The largest percentage of children (37%) were from Anantapur district in Andhra Pradesh state, followed by 17% from Tumkur district in Karnataka state (Figure 4). Nearly 48% of patients were referred from another hospital, and 34% received anti-infective medications before being admitted to the study hospital. The median duration of illness before admission to the study hospital was 6 (IQR 4–9.5) days.

**Table 4:** Demographic details of children with scrub typhus manifesting as acute encephalitis syndrome, southern India

Variable	No. (%) patients
Age, y, n = 87	
<2	2 (2.3)
2–9	50 (57.5)
10–18 y	35 (40.2)
Sex, n = 87	
M	52 (59.8)
F	35 (40.2)
State, n = 81	
Karnataka	44 (54.3)
Andhra Pradesh	36 (44.4)
Tamil Nadu	1 (1.2)
Setting, n = 82	
Rural	64 (78)
Urban	18 (22)
Risk factors, n = 78*	
Contact with shrubs, vegetation, or agricultural farms	49 (62.8)
Contact with animals, birds, or pets	47 (60.3)
Proximity to forest	14 (17.9)

*Based on response to a structured questionnaire in the form of Yes or No.

All 87 children experienced fever and change in mental state; fever was the first symptom in 95% of cases. Around 62% of children had seizures; generalized tonic-clonic seizures were the most common type (74%), and some patients also had focal, tonic, or absence seizures. Upon examination at the time of hospital admission, 55 (64%) patients had altered mental state. The Glasgow Coma Scale at admission ranged from 3 to 15; the median was 13 (IQR 10–15) (Table 5). Signs of meningeal irritation were detected in 48% of patients, cerebellar signs in 21%, and papilledema in 20%. Other neurologic findings were cranial nerve abnormalities (6%), involuntary movements (9%) and photophobia (9%), abnormal tone (50%), decreased power (19%), and abnormal plantar reflexes (24%) (Table 5). Approximately 39% of the patients met criteria for multiorgan dysfunction syndrome (Appendix Table 5).

**Table 5:** Clinical findings of children with scrub typhus manifesting as acute encephalitis syndrome, southern India

Clinical features, n = 86*	No. (%) patients
Duration of illness, d	
<5	38 (44)
>5	48 (56)
Clinical signs and symptoms	
Fever	87 (100)
Change in mental status†	87 (100)
Seizure	53 (61.6)
Vomiting	46 (53.5)
Headache	32 (37.2)
New abnormal speech (e.g., slurred) including the inability to speak	24 (27.9)
Change in personality or behavior	18 (20.9)
Limb weakness	11 (12.8)
Arthralgia or myalgia	7 (8.1)
Respiratory symptoms‡	8 (9.3)
Gastrointestinal symptoms§	29 (33.7)
Urinary symptoms: decreased urine output, burning micturition	3 (3.5)
General and systemic examination findings	
Pallor	23 (26.7)
Icterus	6 (7)
Lymphadenopathy: cervical, inguinal, axillary, mesenteric	16 (18.6)
Edema: periorbital, facial, lower limbs, upper limbs	17 (19.8)
Conjunctivitis/subconjunctival hemorrhage	17 (19.8)
Skin rash	12 (14)
Eschar: axilla, groin, dorsal aspect of penis	4 (4.7)
Abnormal bleeding: nasal, anal, gums	3 (3.5)
Respiratory system findings¶	21 (24.4)
Gastrointestinal system findings#	50 (58.1)
Cardiac system abnormalities: abnormal heart sounds, abnormal pulse	4 (4.7)
Neurologic findings	
Cranial nerve abnormality: 6th and 7th	5 (5.7)**
Sign of meningeal irritation: nuchal rigidity, Kernig’s sign, Brudzinski sign, bulging of anterior fontanelle in infants	41 (47.7)
Photophobia	8 (9.3)
Papilledema	17 (19.8)
Abnormal tone	43 (50)
Paresis/paralysis: decreased power in >1 limbs	16 (18.6)
Exaggerated reflexes	4 (4.7)
Abnormal Plantar reflex	21 (24.4)
Involuntary movements††	8 (9.3)
Cerebellar sign(s)‡‡	18 (20.9)

*All 87 children had fever and change in mental status, but detailed clinical findings of just 86 children were recorded. †Change in mental status was defined as >1 of the following: change in cognition (such as confusion or disorientation), drowsiness, coma, lethargy, irritability, reduced activity, poor feeding, irrelevant/abnormal talk. ‡Includes cough and/or difficulty breathing. Both were present in 5 patients each. §Includes >1 of the following symptoms: abdominal pain, abdominal distension, and diarrhea. Individually, abdominal pain was a symptom in 19 (22%), abdominal distention in 12 (14%), and diarrhea in 3 (3.5%) patients. ¶Signs of respiratory distress occurred in 16 (19%) patients; reduced air entry or abnormal respiratory sounds occurred in 5 (5%) patients. #Hepatomegaly was present in 47 (54.7%) patients, splenomegaly was present in 16 (18.6%) patients, and ascites was present in 4 (4.7%) patients. **Four persons had abnormalities in 6th cranial nerve, and 1 in 7th cranial nerve. ††Opsoclonus and myoclonus, choreoathetoid movements and hemiballismus, abnormal perioral movements, lip smacking, teeth grinding, and rapid eye blinking occurred in 1 patient each; tremors occurred in 2 patients. ‡‡Includes >1 of the following: truncal ataxia, gait abnormality, finger-nose incoordination, nystagmus, dysdiadochokinesis, and dysarthria.

### Laboratory Findings 

Anemia, leukocytosis, thrombocytopenia, transaminitis, hypoalbuminemia, and uremia were each present in >50% of patients (Table 6). CSF results revealed lymphocytic pleocytosis and elevated protein concentration in most patients (Appendix Table 6).

**Table 6:** Laboratory results of children with scrub typhus manifesting as acute encephalitis syndrome, southern India

Sample type and variables	No. (%) patients
Peripheral blood, n = 87 unless stated otherwise	
Anemia	79 (90.8)
Leukocytosis	45 (51.7)
Leukopenia	4 (4.6)
Relative neutrophilia	31 (35.6)
Relative neutropenia	40 (46)
Relative lymphocytosis	43 (49.4)
Relative lymphopenia	26 (29.9)
Thrombocytopenia	45 (51.7)
Hyperbilirubinemia, n = 75	21 (28)
Elevated transaminases, n = 85*	72 (84.7)
Hypoalbuminemia, n = 80	66 (82.5)
Elevated urea, n = 80	56 (70)
Elevated creatinine, n = 86	6 (7)
Cerebrospinal fluid, n = 85 unless stated otherwise	
Pleocytosis†	59 (69.4)
Lymphocytic pleocytosis‡	54 (63.5)
Neutrophilic pleocytosis§	5 (5.8)
Elevated protein, n = 83	51 (61.4)

*Includes elevated levels of aspartate transaminase or alanine transaminase. †Cerebrospinal fluid pleocytosis was recorded if white blood cell counts were >10 cells/μL in infants (1 mo–12 mo of age) and >4 cells/μL in older children (*22*). ‡Cerebrospinal fluid lymphocytic pleocytosis was defined as >50% mononuclear cells in cerebrospinal fluid of patients with pleocytosis (*23*). §Cerebrospinal fluid neutrophilic pleocytosis was defined as a neutrophil count >50% of total leukocytes in patients with cerebrospinal fluid pleocytosis (*23*).

### Treatment

Of the patients with scrub typhus, 44 (51%) required care in the intensive care unit during their hospitalization, and 26 of those required ventilatory support. All patients except 1 were prescribed doxycycline (100 mg 2×/d for 10 days). One patient died during hospitalization.

## Discussion

Our findings suggest that scrub typhus is a major cause of AES in children in southern India. Of 193 (51%) patients with a known etiology, a microbiological test for *O. tsutsugamushi* was positive in 87 (45%) patients, making it the most common etiology obtained in the study. An increasing number of studies in Asia have reported the contribution of *O. tsutsugamushi* to the burden of acute febrile illness in the continent, including South Korea, Japan, China, Taiwan, Thailand, and Bhutan, countries where scrub typhus is a notifiable disease (*24*). Studies including screening for *O. tsutsugamushi* as part of systematic surveillance of childhood CNS infections in Cambodia, Vietnam, Laos, Myanmar, and Thailand report its presence in 1%–4.7% of children (*17*,*25*–*28*). Although studies in India have documented meningoencephalitis as a manifestation of scrub typhus in children (*2*,*29*,*30*), our study highlights the importance of systematic screening for scrub typhus in children with AES in southern India. Scrub typhus is a well-recognized cause of acute febrile illness in the major southern Indian states of Andhra Pradesh and Karnataka (*31*–*33*), but we report scrub typhus is also a common cause of AES in children from these states.

Given the challenges in clinical diagnosis (*10*,*14*,*15*) and complexity of defining the causal relationship of scrub typhus with AES on the basis of serum IgM ELISA, the most widely used test for scrub typhus (*15*), we used a causality strategy. This diagnostic strategy helped in differentiating the certainty of association of 87 AES–scrub typhus cases into 16 cases with confirmed association, 55 with probable association, and 16 with possible association. Real-time PCR, which is confirmatory for scrub typhus, was positive in 6/39 (15%) cases with microbiological evidence of another pathogen and increased the diagnostic association from possible to confirmed. We were able to diagnose scrub typhus in 1 extra case in which IgM ELISA for *O. tsutsugamushi* and tests for other pathogens were negative. Despite systematic testing, the prevalence of positive real-time PCR in children with AES caused by scrub typhus was low in our study (16 [18%] children), although still higher than in other studies (*7*,*34*). PCR positivity might be maximized by collecting clinical samples sooner after illness onset and using whole blood or buffy coat instead of serum to capture intracellular bacteria (*14*,*16*). In this study, patients with a positive PCR had a median duration of illness of 9 (IQR 5.75–12.25) days before clinical specimen sampling versus 11 (IQR 8.5–14.5) days for patients with a negative PCR result.

Because IgM does not ordinarily cross the blood–CSF barrier, presence of those antibodies in CSF implies their production within the CNS (*35*) and higher certainty of association with the infection compared to serum IgM. Using CSF IgM ELISA increased the certainty of association from possible to probable in 23 patients who had simultaneous evidence of another pathogen. Although the kit is recommended for detecting IgM in serum samples only, Murhekar et al. (*6*) observed good correlation between OD values for *O. tsutsugamushi* IgM in serum and CSF. They determined a cutoff OD value of 0.22 after testing CSF samples from 374 children <14 years of age with AES in Gorakhpur, Uttar Pradesh state, India (*35*). A cutoff OD value for IgM in CSF has not been determined in the southern states in India, so we used a higher cutoff (0.5), as used by Behera et al. (*21*) for CSF of children with scrub typhus ME in eastern India.

Our results demonstrate that, compared with PCR, IgM ELISA of CSF had a sensitivity of 92.9%, but with a wide 95% CI, suggesting the estimate is less precise. Although the comparison is indirect, that sensitivity is similar to that of serum IgM by the same ELISA kit (92.4%) used for patients with acute febrile illness caused by scrub typhus in southern India (*14*). The specificity of CSF IgM ELISA was moderate compared to PCR at 82%. That finding might be because PCR positivity was less common in our study, which could be explained by delayed sampling during the course of illness, resulting in a higher likelihood of detection of IgM than DNA. In addition, the use of a single reference standard (PCR) in our study could result in a low PPV of IgM ELISA of CSF. The sensitivity of CSF IgM in patients with scrub typhus ME was 85.7%. Because only 11 patients did not have features suggestive of ME, ascertaining the true specificity is difficult.

Almost three quarters of the patients with AES-scrub typhus had meningoencephalitis. Distinguishing patients with scrub typhus ME from patients with encephalopathy with other causes is crucial. Therapeutic failure of doxycycline, the drug of choice for scrub typhus, has been reported in patients with scrub typhus ME (*36*). This failure could be caused by inadequate concentration of doxycycline in CSF at conventional doses and might indicate the need for increased dosages, intravenous administration, or administration of other antimicrobial agents such as rifampin that have good penetration to the CNS. However, the efficacy of this treatment is yet to be proven (*37*,*38*).

The neurologic manifestations in children with scrub typhus that meet the broader epidemiologic definition of AES are rarely reported (*13*,*25*,*39*,*40*), and no data from southern India have been published. Of all children with scrub typhus in our study, 8 (9%) had involuntary hyperkinetic movements that are rare neurologic manifestations of scrub typhus more often reported in adults than children (*41*). Opsoclonus-myoclonus, best recognized as part of opsoclonus-myoclonus-ataxia syndrome associated with neuroblastoma in children, is rarely caused by infections (*13*,*41*). Only 2 such cases of scrub typhus associated with pediatric opsoclonus-myoclonus-ataxia syndrome have been reported from India (*42*,*43*). Cerebellar signs, which are uncommon in children with scrub typhus (*3*,*13*), were noted in almost one fifth of the children in our study. As reported by Vishwanath et al. (*30*), the sixth cranial nerve was the most affected cranial nerve. Papilledema was detected in 20% of children in our study. Few studies have reported direct retinal involvement and isolated optic disc edema in the absence of raised intracranial pressure in scrub typhus (*29*,*44*,*45*); however, findings in this area remain inconclusive in our study. Presence of eschar typically occurs in 4%–46% of patients with scrub typhus; therefore, while specific, eschar is not a sensitive marker (*30*), and it was found in only 5% of patients in this study.

The first limitation of our study is that, whereas serum IgM ELISA is the most widely used specific test for *O. tsutsugamushi*, we used a single-positive IgM result as a criterion for diagnosis of scrub typhus. Obtaining serial blood samples and performing immunofluorescence or similar assays to demonstrate a 4-fold rise in antibody titers would have enabled more certainty in the diagnosis, especially in cases in which antibodies to another pathogen were detected. However, we defined those patients as having possible scrub typhus to allow for this uncertainty, and they comprised only 18% of the scrub typhus patients in this study. Also, for IgM detection in CSF, we relied on a cutoff value widely used for serum IgM, because a cutoff value for CSF has not been determined in this region. Furthermore, we could not perform sequencing of the PCR-amplified nucleic acid or characterization of surface antigen because of limited resources.

In this study, despite limited accessibility and shortcomings of reference standard tests, we present a stepwise approach to identify scrub typhus as a probable or confirmed etiology by using tests that are relatively easy to access and perform. Our findings highlight the importance of systematic routine testing for the treatable and common pathogen *O. tsutsugamushi* in all patients with AES in southern India, as is practiced in several states in northern India. This testing could have a notable effect on the approach to clinical management and public health interventions for patients with AES. Apart from reinforcing common clinical, epidemiologic, and laboratory findings reported by other studies (*13*,*29*,*39*,*40*,*46*), we report insights into the neurologic spectrum of scrub typhus in children, which appears to be broad and underreported.

Finally, CSF IgM ELISA is a promising test for patients with AES caused by scrub typhus, which requires evaluation in a larger population and determination of a region-specific cutoff OD value. Combining CSF PCR with CSF IgM ELISA wherever feasible might increase the certainty of association between AES and scrub typhus.

Appendix Additional information about association of scrub typhus in children with acute encephalitis syndrome and meningoencephalitis, southern India
