# Supplementary material for: Association of Scrub Typhus in Children with Acute Encephalitis Syndrome and Meningoencephalitis, Southern India
Source: Emerg Infect Dis. 2023 Apr;29(4):711–22. doi: 10.3201/eid2904.221157 (PMC10045701; doi:10.3201/eid2904.221157)
Supplement: Appendix — Additional information about association of scrub typhus in children with acute encephalitis syndrome and meningoencephalitis, southern India [file 22-1157-Techapp-s1.pdf]

Article DOI: https://doi.org/10.3201/eid2904.221157

Association of Scrub Typhus in Children with Acute Encephalitis Syndrome and Meningoencephalitis, Southern India

Appendix

Appendix Table 1. Eligibility criteria for patients in the study

Criteria	Definition
Inclusion criteria	Pediatric patient from 1 mo to 18 y old, at any time of year with acute onset of fever and a change in mental status (including symptoms such as confusion, disorientation, coma, inability to talk, increase in irritability, somnolence or abnormal behavior greater than that seen with usual febrile illness) AND/OR new onset of seizures (excluding simple febrile seizures) AND duration of illness of less than 30 d at presentation
Exclusion criteria	1. Neonates (Age </ = 28 d); 2. Immunocompromised patients; 3. Patient with confirmed non-infectious causes of AES- Autoimmune, Metabolic, Toxin

Appendix Table 2. Co-positive cases*

S.no	Other pathogen(s) identified	Serum IgM against *Orientia tsutsugamushi*	Real time PCR for *Orientia tsutsugamushi*	CSF IgM against *Orientia tsutsugamushi*	Association of AES with Scrub typhus
1	Chikungunya	Positive	Positive	Positive	Confirmed
2	Chikungunya	Positive	Negative	Positive	Probable
3	*Leptospira* species	Positive	Negative	Negative	Possible
4	Chikungunya	Positive	Positive	Positive	Confirmed
5	Chikungunya	Positive	Negative	Positive	Probable
6	JEV	Positive	Negative	Negative	Possible
7	Chikungunya	Positive	Negative	Negative	Possible
8	Chikungunya	Positive	Positive	Positive	Confirmed
9	Chikungunya	Positive	Negative	Positive	Probable
10	Chikungunya	Positive	Negative	Positive	Probable
11	Dengue & Chikungunya	Positive	Negative	Positive	Probable
12	Chikungunya & *Leptospira* species	Positive	Negative	Positive	Probable
13	JEV, Dengue, Chikungunya	Positive	Negative	Negative	Possible
14	*Leptospira* species	Positive	Negative	Positive	Probable
I5	*Leptospira* species	Positive	Negative	Positive	Probable
16	Chikungunya	Positive	Negative	Negative	Possible
17	Chikungunya	Positive	Negative	Negative	Possible
18	JEV	Positive	Negative	Negative	Possible
19	Chikungunya	Positive	Negative	Positive	Probable
20	Dengue, Chikungunya, *Leptospira* species	Positive	Negative	Negative	Possible
21	Chikungunya	Positive	Positive	Positive	Confirmed
22	*Leptospira* species	Positive	Negative	CSF not received	Possible
23	Dengue	Positive	Negative	Positive	Probable
24	JEV, Dengue, Chikungunya	Positive	Negative	Positive	Probable
25	Chikungunya	Positive	Negative	Positive	Probable
26	Dengue	Positive	Positive	Positive	Confirmed
27	JEV, Dengue, Chikungunya	Positive	Negative	CSF not received	Possible
28	JEV, Dengue, Chikungunya	Positive	Negative	Positive	Probable
29	Chikungunya	Positive	Negative	Positive	Probable
30	Chikungunya	Positive	Positive	Positive	Confirmed
31	JEV, Dengue, Chikungunya	Positive	Negative	Positive	Probable

S.no	Other pathogen(s) identified	Serum IgM against *Orientia tsutsugamushi*	Real time PCR for *Orientia tsutsugamushi*	CSF IgM against *Orientia tsutsugamushi*	Association of AES with Scrub typhus
32	Chikungunya	Positive	Negative	Negative	Possible
33	Chikungunya	Positive	Negative	Negative	Possible
34	JEV, Dengue, Chikungunya	Positive	Negative	Negative	Possible
35	Chikungunya	Positive	Negative	Positive	Probable
36	Chikungunya	Positive	Negative	CSF not received	Possible
37	Dengue	Positive	Negative	Negative	Possible
38	JEV, Dengue, Chikungunya	Positive	Negative	Positive	Probable
39	Varicella Zoster virus	Positive	Negative	Negative	Possible

*AES, acute encephalitis syndrome; JEV, Japanese encephalitis virus.

Appendix Table 3. Patients with meningoencephalitis (n = 65)

CSF Pleocytosis	MRI Brain suggestive of ME	CSF PCR Positive	Total (65)
+	+	+	1
+	+	-	7
+	-	+	12
+	-	-	39
-	+	+	1
-	+	-	4
-	-	+	1

Appendix Table 4. Association of scrub typhus in patients with and without meningoencephalitis (n = 87)

Suggestive of meningoencephalitis	Criteria	Association with scrub typhus	CSF IgM	No. of patients
Yes, n = 65	CSF Pleocytosis n = 59	Confirmed	Positive	13
		Probable (Single)	Positive	21
		Probable (Single)	Negative	4
		Probable (Single)	CSF not received	1
		Probable (Co-pos)	Positive	13
		Possible (Co-pos)	Negative	6
		Possible (Co-pos)	CSF not received	1
	CSF real-time PCR Positive n = 2	Confirmed	Positive	1
		Confirmed	Negative	1
	MRI Brain positive n = 4	Probable (Single)	Positive	1
		Probable (Single)	Negative	1
		Possible (Co-pos)	Negative	2
No, n = 22	None	Confirmed	Negative	1
		Probable (Single)	Positive	5
		Probable (Single)	Negative	5
		Probable (Co-pos)	Positive	4
		Possible (Co-pos)	Negative	5
		Possible (Co-pos)	CSF not received	2

Appendix Table 5. Organ dysfunction in children with scrub typhus manifesting as acute encephalitis syndrome

Organ dysfunction	No. of patients in whom dysfunction could be assessed	No. patients with dysfunction (%)
1 or more organ dysfunction present	87	58 (66.67)
Neurologic	84	27 (31.03)
Hematological	87	26 (29.89)
Hepatic	87	36 (41.38)
Respiratory	86	28 (32.18)
Multi-organ dysfunction	87	34 (39.08)

Appendix Table 6. CSF analysis of children with scrub typhus manifesting as acute encephalitis syndrome

CSF Analysis	No. patients (N)	Median (IQR, Q1-Q3)
CSF total cells/μL	85	12 (4–35)
CSF lymphocytes/μL	85	11.5 (3.25–27.75)
CSF neutrophils/μL	85	0 (0–2)
CSF protein (mg/dL)	83	56.5 (37.15–90)
CSF glucose (mg/dL)	83	56.9 (50.45–64.75)

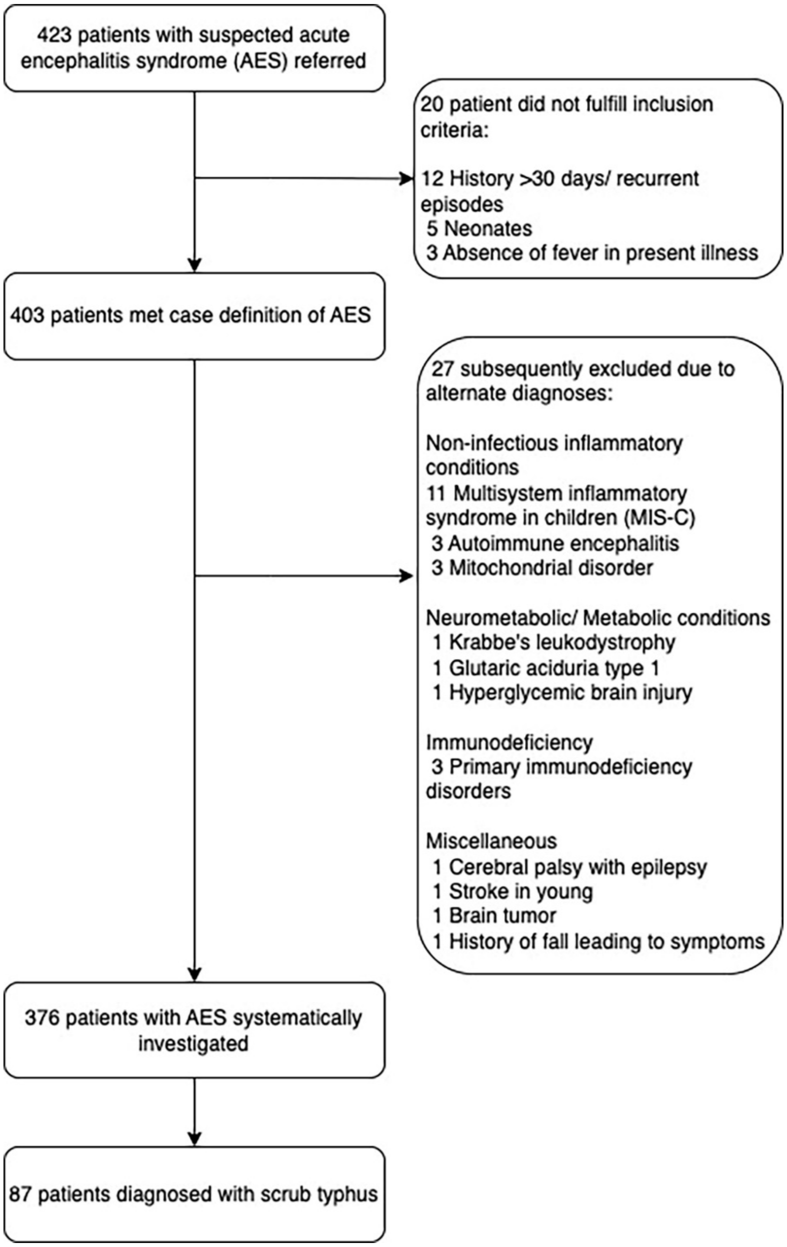

Appendix Figure 1. Recruitment strategy of patients in our study

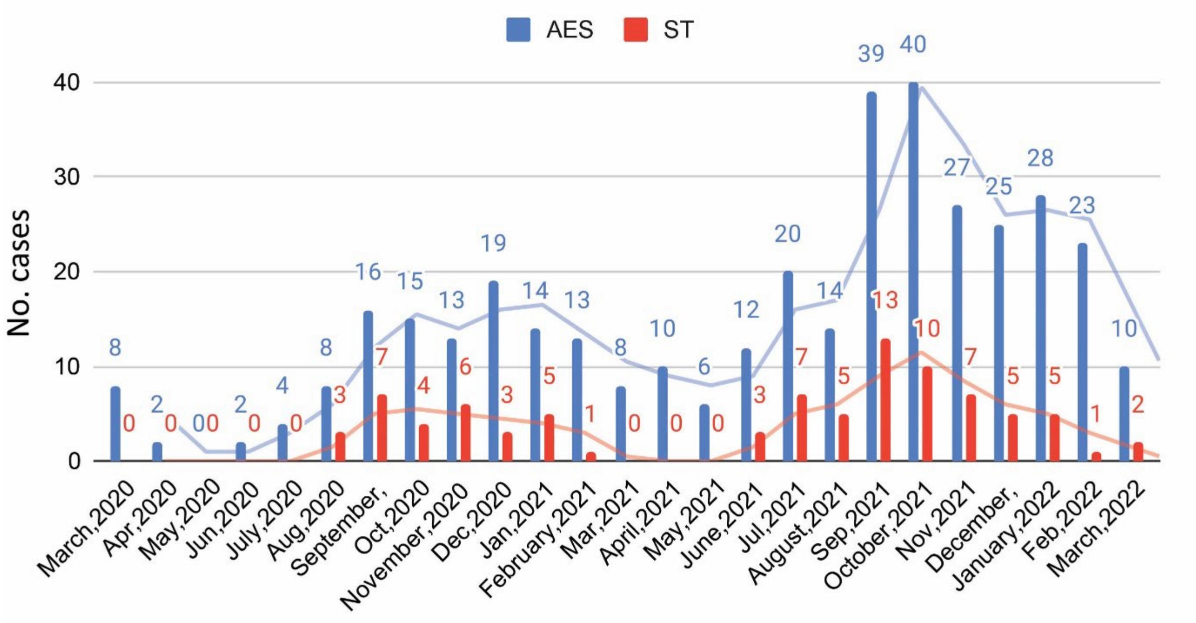

Appendix Figure 2. Number of childhood AES and AES-ST cases per month in the study period.

AES, acute encephalitis syndrome; AES-ST, scrub typhus manifesting as acute encephalitis syndrome.

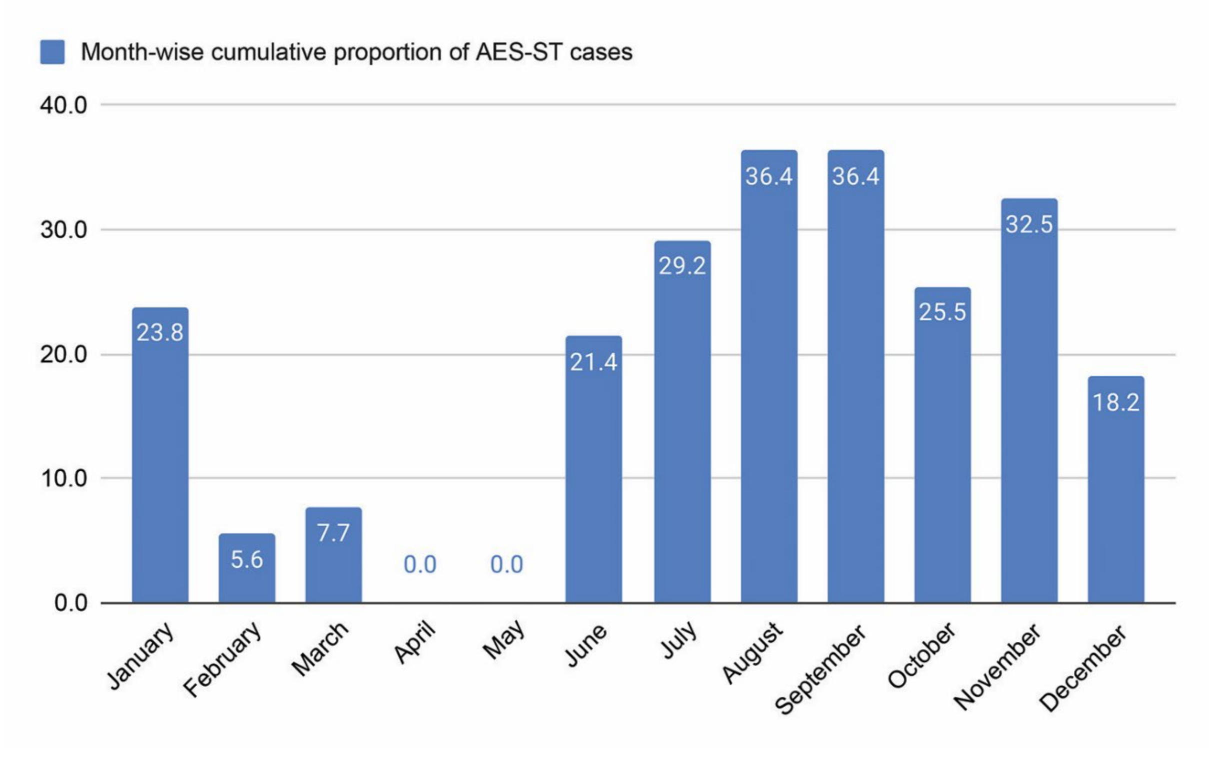

Appendix Figure 3. Month-wise cumulative proportion of AES-ST cases in our study
